# Serious infections in ANCA-associated vasculitides in the biologic era: real-life data from a multicenter cohort of 162 patients

**DOI:** 10.1186/s13075-021-02452-8

**Published:** 2021-03-20

**Authors:** Konstantinos Thomas, Evangelia Argyriou, Noemin Kapsala, Alexandros Panagiotopoulos, Aglaia Chalkia, Emilia Hadziyannis, Kyriaki Boki, Pelagia Katsimbri, Dimitrios T. Boumpas, Panagiota Giannou, Dimitrios Petras, Dimitrios Vassilopoulos

**Affiliations:** 1 Joint Rheumatology Program, Clinical Immunology-Rheumatology Unit, 2nd Department of Medicine and Laboratory, National and Kapodistrian University of Athens School of Medicine, Hippokration General Hospital, 114 Vass. Sophias Ave, 115 27 Athens, Greece; 2 Rheumatology Unit, Sismanoglio General Hospital, Athens, Greece; 3 Joint Rheumatology Program, Clinical Immunology-Rheumatology Unit, 4th Department of Medicine, National and Kapodistrian University of Athens School of Medicine, Athens, Greece; 4 Nephrology Department, Hippokration General Hospital, Athens, Greece

**Keywords:** ANCA vasculitis, Infections, Rituximab

## Abstract

**Background:**

Serious infections (SI) are common in patients with ANCA-associated vasculitides (AAV) like granulomatosis with polyangiitis (GPA) and microscopic polyangiitis (MPA). Real-life data regarding their incidence and predisposing factors—after the introduction of B cell depleting agents—are limited while data quantifying the risk per treatment modality and year of the disease are missing. Here, we aim to describe in details the incidence and the risk factors for SI in a contemporary AAV cohort.

**Methods:**

Multicenter, observational, retrospective study of AAV patients followed in three tertiary referral centers.

**Results:**

We included 162 patients with GPA (63%) and MPA (37%), males 51.9%, mean age 60.9 years, ΑΝCA+ 86%, and generalized disease 80%. During follow-up (891.2 patient-years, mean 5.4 years), 67 SI were recorded in 50 patients at an incidence rate of 7.5 per 100 patient-years. The SI incidence rate was higher during induction with cyclophosphamide (CYC) compared to rituximab (RTX, 19.3 vs. 11.3 per 100 patient-years, respectively) while it was lower and comparable between RTX and other regimens (5.52 vs. 4.54 per 100 patient-years, respectively) in the maintenance phase. By multivariate analysis, plasmapheresis (PLEX) and/or dialysis was a strong predictor for an SI during the 1st year after diagnosis (OR = 3.16, 95% CI 1.001–9.96) and throughout the follow-up period (OR = 5.21, 95% CI 1.93–14.07). In contrast, a higher baseline BVAS (OR = 1.11, 95% CI 1.01–1.21) was associated with SI only during the 1st year.

**Conclusions:**

In this real-life study of patients with AAV, the SI incidence was higher during CYC compared to RTX induction while there was no difference between RTX and other agents used for maintenance therapy. Higher disease activity at baseline and need for PLEX and/or dialysis were independent factors associated with an SI.

**Supplementary Information:**

The online version contains supplementary material available at 10.1186/s13075-021-02452-8.

## Background

Anti-neutrophil cytoplasmic antibody (ANCA)-associated vasculitides (AAV) are among the rheumatic diseases with the highest mortality and morbidity, due to their major multi-organ involvement (lungs, kidneys) and their relapsing nature requiring aggressive immunosuppressive treatment [1, 2]. Numerous studies from several registries have shown that serious infections (SI) contribute significantly to the increased disease mortality, especially early in the disease course (1st year after diagnosis) [3].

Despite the substantial progress in the AAV therapeutics with the introduction of monoclonal biologic therapies (rituximab-RTX) [4, 5], the incidence of SI in AAV has remained relatively stable. Their rate is significantly higher compared to the age-matched general population [6] without decreasing trends in recent cohorts [7]. Although long-term extension studies from the initial randomized controlled trials (RCTs) have provided useful data regarding the incidence and risk factors for SI in these patients, real-world data for the different phases of AAV comparing SI in the biologic era are limited.

The aim of our study was to describe the incidence, type and risk factors for SI as well as their association with disease status and treatment in AAV patients in real-world settings.

## Methods

### Patients

We conducted a multicenter, observational, retrospective study of patients with AAV followed in three referral centers in the Athens Metropolitan, Greece (Hippokration General Hospital—HGH, Attikon University Hospital—AUH, and Sismanoglion General Hospital—SGH). The study was approved by the Institutional Review Board (57/26-3-2018).

Inclusion criteria were age ≥ 18 years, fulfillment of the Revised International Chapel Hill Definitions for granulomatosis with polyangiitis (GPA) and microscopic polyangiitis (MPA), and a minimum of 3 months of follow-up after induction of remission initiation. Patients with eosinophilic granulomatosis with polyangiitis (EGPA) were excluded due to the different natural history, in order to achieve a more homogenous study population.

The following data were collected for each participant: patient and disease characteristics (age, sex, date of diagnosis, diseases severity, disease activity at baseline (BVAS v.3), ANCA serology, organ involvement, relapses, renal function [estimated glomerular filtration rate (eGFR) by CKD-EPI formula] during first induction of remission course) and treatment patterns (treatment types and duration for each treatment, both for induction and maintenance of remission as well as initial glucocorticoids (GC) dose at diagnosis). The choice, dosing, and duration of each treatment were upon physicians’ discretion according to the most recent National and International Recommendations. Where indicated according to induction treatment type, the chemoprophylaxis against *Pneumocystis jirovecii* was also recorded. Comorbidities at diagnosis (chronic obstructive pulmonary disease, cardiovascular disease, stroke, hypertension, fracture, depression, diabetes, gastric ulcer) as well as the composite Rheumatic Disease Comorbidity Index (RDCI) were also calculated, as described elsewhere [8].

### Serious infections

We defined SI as those needing hospitalization or intravenous antibiotics as well as opportunistic infections. Given the high frequency of herpes zoster (HZ) in AAV population and the accompanying morbidity of this infection, all cases of HZ were considered as SI irrespective of hospitalization need.

### Drug exposure

We estimated the exposure period in patient-years for all patients to any of the following treatment regimens: cyclophosphamide (CYC), RTX as induction of remission, RTX as maintenance of remission, and any other maintenance treatment. Induction of remission drug exposure with CYC or RTX included both the initial treatment at diagnosis as well as the treatment of relapses. For CYC, the exposure period was the time interval between treatment initiation and 3 months after the last dose while for RTX the exposure period was defined as the time interval between first dose and 6 months after the last dose. Induction CYC and RTX exposure periods included both the induction at diagnosis and at relapses. For all other treatments, the exposure period was started and ended with the first and last dose. Incidence rates for each particular drug exposure were calculated by dividing the number of SI during the exposure with the patient-years of each exposure.

### Statistical analysis

We performed the statistical analyses with SPSS (IBM SPSS Statistics for Windows, v. 23.0. Armonk, NY: IBM Corp), OpenEpi, and Microsoft Office Excel 2013. Dichotomous variables are presented as percentages while continuous variables are presented as mean (standard deviation) for normal and median (interquartile range) for nonparametric distributions, respectively. Chi-square was used for comparison of dichotomous and Mann-Whitney or *t* test for continuous variables. The threshold of statistical significance was set as *p* value < 0.05. Free-of-infection survival (FIS) was evaluated with Kaplan-Meier analysis, and log rank test was implemented to compare FIS among several subgroups. Patients were censored at the time of serious infection or at the end of follow-up period (last available visit).

Univariate and multivariate logistic regression was performed in order to identify risk factors for SI during the total follow-up and during 1st year. Variables with *p* < 0.05 in univariate analysis were included in the multivariate model (backwards selection) and those with *p* < 0.05 were retained until the final stage of the model.

## Results

### Patient characteristics at diagnosis

One hundred sixty two (162) patients seen between January 1990 and May 2020 were included in the study; the majority of them were included after 2010 (118/162, 73%); 51.9% of patients were males, with a mean age of 60.9 ± 15.7 years (Table 1). The majority of patients had generalized disease (79.6%) and GPA (63%). ANCA serology was available in 159 patients with 44% being positive for C-/PR3-ANCA, 43.4% for P-/MPO-ANCA, and 12.6% being ANCA negative. The mean eGFR at diagnosis was 59.1 mL/min with approximately one quarter of patients (23.5%) having values < 30 mL/min. Mean BVAS at diagnosis was 12.75 ± 6.25 with 23 (14.2%) and 10 (6.2%) of patients needing renal replacement therapy and/or plasma exchange (PLEX) at diagnosis, respectively.

**Table 1:** Patient and disease characteristics

Variable	Results
**Males,** ***n*** **(%)**	84 (51.9%)
**Age at diagnosis, years, mean ± SD**	60.9 ± 15.7
**Follow-up period, years, mean ± SD**	5.4 ± 5.0
**AAV type (*n*, %)**	
GPA	102 (63%)
MPA	60 (37%)
**ANCA,** ***n*** **(%),** ***n*** **= 159**	
C-/PR3-ANCA	70 (44%)
P-/MPO-ANCA	69 (43%)
Negative	20 (13%)
**Severity,** ***n*** **(%)**	
Generalized	129 (80%)
Localized	33 (20%)
**Organ involvement**	
Lung	125 (77%)
Kidney	114 (70%)
Joints	78 (48%)
ENT	71 (44%)
Nervous	40 (25%)
Skin	37 (23%)
Mucosal/eyes	25 (15%)
Other	33 (20%)
**BVAS at diagnosis, mean ± SD**	12.75 ± 6.25
**Dialysis,** ***n*** **(%)**	23 (14.2%)
**PLEX,** ***n*** **(%)**	10 (6.2%)
**Initial treatment (*n*, %)**	
CYC	99 (61%)
RTX	29 (18%)
MTX	13 (8%)
MMF	3 (2%)
RTX+CYC	9 (6%)
GC only	9 (6%)
Maintenance therapy after initial induction* (*n*, %)	
AZA	44 (38.1%)
RTX	43 (37.5%)
MMF	16 (13.9%)
MTX	10 (8.7%)
Other	2 (1.7%)
**Comorbidities (*n*, %)**	
Arterial hypertension	70 (43%)
Diabetes	30 (18%)
Cardiovascular disease (CVD)	27 (17%)
Depression	17 (10%)
Chronic obstructive pulmonary disease (COPD)	11 (7%)
Fracture	9 (6%)
Gastric ulcer	7 (4%)
Stroke	3 (2%)

*Data from *n* = 115 patients treated with CYC, RTX, or CYC/RTX during initial induction of remission, after excluding those not at remission at the end of induction (*n* = 6), who did not take any therapy (*n* = 1), not had completed induction at last visit (*n* = 8), and missing data regarding the type of maintenance treatment (*n* = 6)

*SD* standard deviation, *AAV* ANCA-associated vasculitis, *ANCA* anti-neutrophil cytoplasmic antibodies, *ENT* ear-nose-throat, *BVAS* Birmingham Vasculitis Activity Score, *PLEX* plasma exchange, *CYC* cyclophosphamide, *RTX* rituximab, *MTX* methotrexate, *MMF* mycophenolate mofetil, *GC* glucocorticoids, *AZA* azathioprine, *CVD* cardiovascular disease, *COPD* chronic obstructive pulmonary disease

Patients were treated with CYC alone (61%), RTX alone (18%), or their combination (9%) in addition to GCs (mean initial daily prednisolone dose 44 ± 15 mg) as induction therapies. Among patients treated with CYC and/or RTX (*n* = 137), 74% (*n* = 102) received chemoprophylaxis against *Pneumocystis jirovecii* pneumonia (PCP). No predictors for PCP prophylaxis were identified (Suppl. Table 1). A comparable percentage of patients were treated with azathioprine (AZA, 38.1%) or RTX (37.5%) as maintenance therapy (Table 1).

### Incidence and type of serious infections

During the follow-up period (891.2 patient-years, mean duration 5.4 years), 50 patients (32%) developed 67 SI with an overall incidence rate of 7.5/100 patient-years. The median time to the 1st infection was 1.1 years (range 0.36–4.14). The mortality rate during the same period was 9.2% (*n* = 15). Almost half of the infections were located in the respiratory tract (45%), followed by HZ (24%), gastrointestinal tract (9%), bacteremia (9%), and urinary tract infections (9%, see Table 2). The median daily prednisolone dose at the time of infection was 19 mg (Table 2).

**Table 2:** Type and treatment at the time of serious infections

	***n*** = 67
**Sites and types of infections,** ***n*** **(%)**	
- Respiratory tract	30 (45%)
- Herpes zoster	16 (24%)
- Gastrointestinal tract	6 (9%)
- Bacteremia	6 (9%)
- Urinary tract	6 (9%)
- ABSSI	2 (3%)
- Other	1 (2%)
**Type of treatment at infection, ** ***n*** **(%)**	
- Induction	29 (43%)
- Maintenance	36 (54%)
- No treatment	2 (3%)
**Prednisone dose at the time of infection, median (range)**	19 (5–30) mg/day

*ABSSI* acute bacterial skin and skin structure infections, *mg* milligrams

### Timing of serious infections

The overall incidence rate of SI and that according to the year after diagnosis is shown in Table 3. Most infections (42%) occurred during the 1st year after diagnosis. The respective SI incidence rate was 18.6, 6.2, 5.7, and 4.7/100 patient-years during the 1st, 2nd, 3rd, and > 4th year after diagnosis (Table 3). The respective incidence rate ratios for each of the first 3 years after diagnosis compared to the incidence of 4th year and beyond as reference were 3.91, 1.31, and 1.20, respectively (Table 3).

**Table 3:** Overall and according to the year after diagnosis serious infection incidence rates

	Overall	1st year	2nd year	3rd year	> 4th year
***n*** of events*	67	28	8	6	24
**%**	100%	42%	12%	9%	36%
**Patient-years**	891.2	150.77	128.38	105.65	506.08
**Incidence rate (per 100 patient-years)**	7.5	18.57	6.23	5.67	4.74
**Incidence rate ratio (95% CI)**	**NA**	**3.91 (2.26–6.81)**	1.31 (0.55–2.85)	1.20 (0.44–2.82)	ref

*Exact time missing in 1 case

*CI* confidence interval, *NA* non-applicable

### Risk factors for SI during

A. The entire follow-up period

The characteristics of patients who developed (*n* = 50) or not (*n* = 112) an SI are shown in Suppl. Table 2. Patients with SI were more likely to have been managed with PLEX and/or dialysis (29% vs. 8.9%, *p* = 0.002) and treated with higher initial prednisolone dose (48.4 ± 16.9 vs. 42.2 ± 13.9 mg/day, *p* = 0.01) and to have lower eGFR (50.9 ± 36 vs. 62.9 ± 34.5 mL/min, *p* = 0.048), higher BVAS at diagnosis (14.6 ± 6.3 vs. 11.9 ± 6, *p* = 0.01), and higher co-morbidity burden [median RDCI (IQR) 1 (0–2) vs. 1 (1–3), *p* = 0.03] compared to those without SI.

**Fig. 1:**
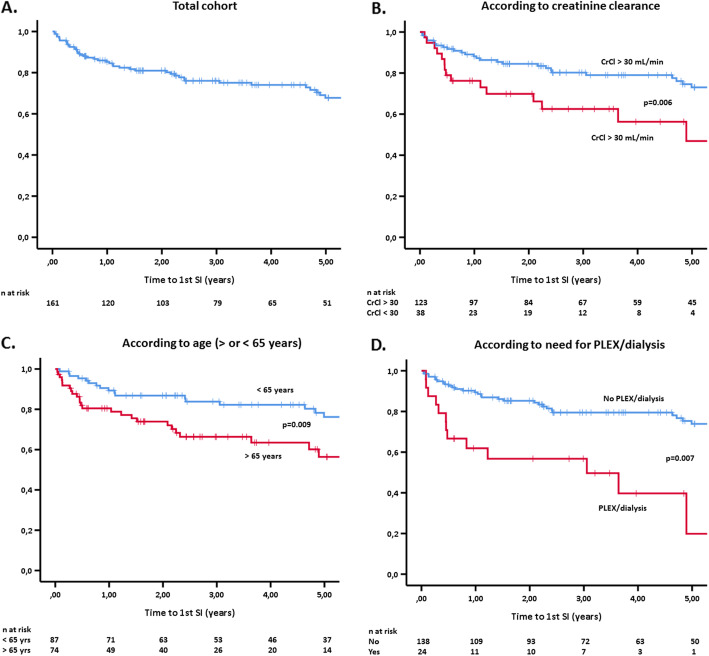
Free-of-infection survival (FIS) curves in the total cohort (**a**) and according to creatinine clearance (**b**), age (**c**), and need for PLEX and/or dialysis (**d**). FIS, free-of-infection survival; PLEX, plasma exchange; CrCl, creatinine clearance; SI, serious infection

By multivariate analysis, patients who had been managed with PLEX and/or dialysis had almost a five times higher risk for developing an SI compared to those who did not (OR = 5.21, 95% CI 1.93–14.07, Suppl. Table 3). Specific risk factors such as impaired renal function at diagnosis (eGFR < 30 mL/min), advanced age, and the need for PLEX and/or dialysis had the greatest impact on SI development as shown in Fig. 1.

B. The 1st year after diagnosis

During the 1st year after diagnosis, 23 patients (14%) developed an SI (Suppl. Table 4). Compared to those who did not, they were older (68.2 ± 13.6 vs. 59.7 ± 15.8 years, *p* = 0.016), more likely to need PLEX and/or dialysis (39% vs 11%, *p* < 0.001) or CYC-RTX combination therapy (19% vs. 3.7%, *p* = 0.008), to have a history of diabetes (43.5% vs. 14.5%, *p* = 0.001), lower eGFR (40.6 ± 36.1 vs. 62.5 ± 34.4 mL/min, *p* = 0.006), higher BVAS (16.7 ± 5.5 vs. 12.1 ± 6.2, *p* = 0.01), and to be given a higher prednisolone dose at diagnosis (51.8 ± 18.5 vs. 42.5 ± 13.9 mg/day, *p* = 0.007). The SI incidence was comparable among those who had received or not PCP prophylaxis (14.6% vs. 19.4%, *p* = 0.52). By multivariate analysis, the need for PLEX and/or dialysis (OR = 3.16, 95% CI 1.001–9.96) and a higher BVAS at diagnosis (OR = 1.11, 95% CI 1.01–1.21) were independent predictors of an SI (Suppl. Table 5).

### Risk for SI during exposure to discrete drug regimens

The potential contributing role of various immunosuppressives in SI risk at different treatment phases (induction of remission, maintenance, off therapy) was also assessed. The incidence of SI was higher during the induction phase compared to the maintenance or off therapy phases (see Table 4). Induction at the time of diagnosis or at relapses with CYC had a higher SI incidence rate compared to RTX (19.34 vs. 11.34/100 patient-years). In order to identify potential confounders for the use of CYC vs. RTX as induction regimens, we compared the patient characteristics between the two groups. Notably, we did not find any statistically significant differences between these groups with the exception of a higher proportion of MPA patients among those treated with CYC (44.4% vs. 24.1%, *p* = 0.049, see Suppl. Table 6). An attempt was made to perform a propensity matched analysis (PMA) between patients treated with CYC or RTX at diagnosis, but the small number of matched patient-pairs (*n* = 28) and the respective SI events (*n* = 7) precluded any reliable statistical comparisons and conclusions (data not shown).

**Table 4:** Serious infections incidence rates according to different treatment phases

	CYC induction	RTX induction	RTX maintenance	Other maintenance agents or off-therapy
***n*** **of events**	21	7	9	25
**Patient-years**	108.6	61.75	162.93	551.07
**Incidence (per 100 patient-years)**	19.34	11.34	5.52	4.53
**Incidence rate ratio (95% CI)**	**4.24** (2.35–7.61**)**	2.49 (0.99–5.57)	1.22 (0.54–2.55)	Ref

*CYC* cyclophosphamide, *RTX* rituximab, *CI* confidence intervals

**Fig. 2:**
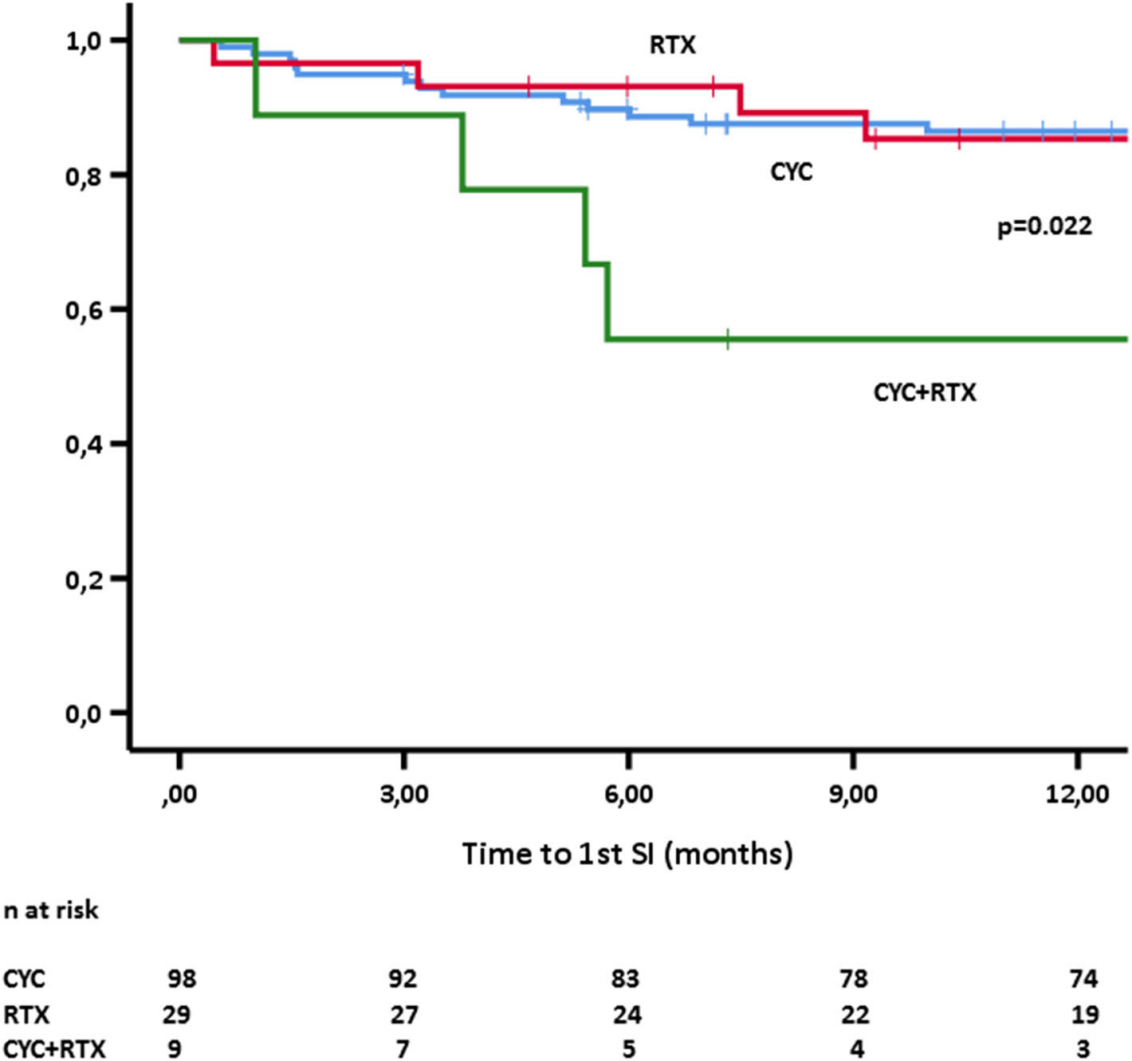
Comparison of free-of-infection survival (FIS) curves during the 1st year after diagnosis according to initial induction of remission regimens. FIS, free-of-infection survival; CYC, cyclophosphamide; RTX, rituximab

Regarding maintenance regimens, there was no difference between patients treated with various immunosuppressives (AZA, MTX, MMF) or being off-therapy (4.53/100 patient-years) and those treated with RTX (5.52/100 patient-years, see Table 4). When the former was used as the reference category, the SI incidence rate ratios for CYC induction, RTX induction, and RTX maintenance therapy were 4.24 (95% CI 2.35–7.61), 2.49 (95% CI 0.99–5.57), and 1.22 (95% CI 0.54–2.55), respectively (Table 4). Patients treated with a more aggressive induction regimen combining CYC and RTX had a higher SI rate compared to CYC or RTX induction alone the 1st year after diagnosis (*p* = 0.022 by log rank, Fig. 2). We did not observe any difference in SI rates between CYC and RTX regimens at the end of the 1st year.

## Discussion

The goals of this study were to thoroughly evaluate the risk factors and incidence for SI in a real-world AAV cohort in different phases of the disease (induction vs. maintenance) treated with various treatment regimens (non-biologics vs. biologics) in the modern era of AAV treatment.

During the 5-year follow-up period, the overall incidence of SI in our AAV patients was 7.5 per 100 patient years which is much lower to the incidence reported in older studies [3, 9]. This decreasing incidence could be attributed to the better overall care of these patients and the limited use of CYC and GCs given for extended periods of time.

Nevertheless, we observed a significant variation of SI incidence according to the time after diagnosis and the treatment regimen used. As expected, the SI incidence rate was the highest during the 1st year after diagnosis (18.57/100 patient-years) with a decreasing trend over time. Compared with the respective SI incidence rate late after diagnosis (4th year and beyond), the risk for SI during the 1st year was approximately 4 times higher. This temporal trend comes in agreement with older [3, 9] and more recently published studies [6, 10].

In regard to the treatment phase, the SI risk was higher during the induction compared to the maintenance phase. Over the last decade, RTX has been established as an alternative to CYC induction agent for severe AAV [11]. However, literature data comparing the SI risk between these two induction regimens are limited and mainly derived from the original RCTs [4, 5]. In the RAVE trial, the SI incidence was similar for the CYC and RTX groups (14/100 patient-years at 6 months) [4], whereas in the RITUXVAS trial where patients with more severe renal disease were included, the incidence rate was slightly higher for CYC compared to RTX (27.2 vs. 21.2/100 patient-years at 12 months) [5].

In our cohort, 61% of patients were treated with CYC, 18% with RTX while 6% received both as induction therapy in combination with GCs. Free-of-infection survival at 12 months after diagnosis did not differ between CYC and RTX. However, after including in the analysis the total induction courses (both at diagnosis and at relapses), we found that CYC-treated patients had a higher SI incidence rate compared to RTX (19.34 vs. 11.34 per 100 patient-years, respectively) although there were no statistically significant differences in their baseline characteristics (except for the higher % of MPA patients in the CYC group). Although MPA patients may have a different clinical phenotype and course, these patients as a group did not have a statistically significant higher risk for SI compared to GPA patients while MPA as a diagnosis was not identified as an independent risk factor for SI in the uni- or multivariate analysis.

Furthermore, we found that patients who had been treated with CYC and RTX combination induction therapy had a higher risk for SI compared to those treated with each agent alone. These findings though should be interpreted with caution since only a small proportion of patients with the most severe disease were treated with this regimen. In a recent retrospective analysis of 239 AAV patients treated with a combination of RTX (1 g q4months for the first 2 years) and CYC (pos for the first 2 months), Cortazar et al. observed a high SI incidence rate during induction (29 per 100 patient-years) [12]. Despite this aggressive regimen and the high SI rate, the mortality rate during the induction period was very low (2%) indicating that early control of disease activity is crucial for survival.

RTX has also emerged as an efficacious maintenance therapy for AAV (after induction with CYC or RTX) compared to other agents, particularly AZA [13], and has been included in the most recent guidelines [14]. RCT data comparing the SI risk between RTX and AZA given for ~ 2 years showed no significant differences between the 2 groups (8 events/58 patients in the AZA group compared to 11 events/57 patients in the RTX group) [13].

Similarly in our cohort where an equal number of patients were treated with AZA or RTX for maintenance of remission, the SI incidence rate was comparable (4.54 vs. 5.52/100 patient-years, respectively). Specifically for RTX maintenance therapy, the SI rate was similar to that reported from a recent meta-analysis of 18 studies including 528 patients (6.5/100 patient-years) [15] and in the RTX arm (7.7/100 patient-years) of the long term extension of the MAINRITSAN2 RCT [16].

When we looked specifically for SI predictive factors during the early and entire follow-up period, two main risk factors emerged: the high baseline disease activity (as expressed by the BVAS) and the early need for PLEX and/or dialysis. Regarding disease activity, SI risk increased by approximately 10% for each 1-point increase of the baseline BVAS. Disease activity, assessed by various indices, was also a predictor for SI in other recent studies [10, 17]. We also found that the need for PLEX and/or dialysis was associated with a 3.4-times increase in the SI risk. A similar 3-fold increase in the risk for bacterial infections with dialysis was also reported by Garcia-Vives et al. [17]. As for the role of PLEX, data from the MEPEX trial have shown that the proportion of deaths attributed to infection was numerically higher in PLEX-treated patients compared to those treated with pulse GCs (43% vs. 26%) [18] as add-on therapies to the usual induction regimens. Nevertheless, the recently published PEXIVAS study did not find any difference in SI incidence between patients with severe disease that received PLEX versus those who did not [19].

The strengths of our study include its real-life multicenter design, the inclusion of patients from referral rheumatology centers with long experience in the care of AAV patients, the exclusion of EGPA patients that resulted in a more homogenous cohort, the long term follow-up of ~ 5.4 years, and the detailed presentation of SI incidence during the different phases of the disease and drug exposures.

Our study has also limitations. The first is its retrospective design that could lead to underestimation of the SI incidence, as some of the infections (especially HZ not needing hospitalization) could have been diagnosed and treated by other specialties. We consider this unlikely since caring rheumatologists in these referral centers are usually responsible for the whole care of patients including treatment of their co-morbidities. Second, there was no pre-specified, unified treatment protocol across participating centers. We believe though that all these referral centers are following the most recent International and Greek Guidelines for AAV treatment. A third significant limitation is the missing total exposure to GCs during follow-up. The correlation between GC doses and SI risk is well described both in earlier [9] and more recent studies [19–21]. Finally, Vasculitis Damage Index (VDI) data were not consistently recorded and disease- and treatment-related damage accumulation could be a confounder in our analysis.

Given the high incidence and the significant mortality of SI in patients with AAV, we believe that further improvement to the direction of early recognition of patients at risk and less toxic therapies is mandatory. Novel biomarkers of humoral and cell-mediated iatrogenic immunodeficiencies, enhancement of vaccination uptake, personalized treatment according to the involved pathways of the disease, and the introduction of GC-sparing regimens could substantially contribute in the reduction of these devastating complications.

## Conclusions

In conclusion, our real life, long-term study shows that the incidence of SI in the modern era of biologics was rather low in AAV patients and predominantly determined by the baseline disease activity, time after diagnosis, and the treatment regimens used. Patients treated with RTX have a slightly lower rate of SI compared to CYC during the induction phase while there was no difference compared to the other regimens during the maintenance phase. These findings indicate that despite the decrease in relapse rates and improved survival achieved by RTX in daily practice, the rate of SI especially during the induction phase remains high. Moreover, in patients with high baseline disease activity, the use of combination schemes with CYC and RTX was associated with a higher SI risk during the 1st year. In the absence of RCTs directly comparing combination schemes of CYC and RTX to each agent alone for induction of remission, caution is needed when this regimen is used. Altogether, our results emphasize the challenges that caring physicians are facing for the treatment of AAV and highlight the unmet needs for safer treatment regimens, especially during the early phases of the disease.

## Supplementary Information

**Additional file 1: Suppl. Table 1.** Comparison of baseline characteristics according to the prescription of chemoprophylaxis against *Pneumocystis jirovecii* during the initial induction of the remission (*n* = 135)*. **Suppl. Table 2.** Comparison between patients who developed (*n* = 50) or not (*n* = 112) serious infections (SI). **Suppl. Table 3.** Uni- and multi-variate logistic regression analysis of factors associated with serious infections (SI). **Suppl. Table 4.** Comparison between patients who developed (*n* = 23) or not (*n* = 138) serious infections (SI) during the 1st year after diagnosis. **Suppl. Table 5.** Uni- and multi-variate logistic regression analysis of factors associated with serious infections (SI) during the 1st year after diagnosis. **Suppl. Table 6.** Comparison of patient characteristics treated with cyclophosphamide (CYC) or rituximab (RTX) based induction regimens.

## Data Availability

The datasets used and/or analyzed during the current study are available from the corresponding author on reasonable request.

## Abbreviations

AAV: ANCA-associated vasculitides
ANCA: Anti-neutrophil cytoplasmic antibodies
AZA: Azathioprine
BVAS v.3: Birmingham Vasculitis Activity Score (version 3)
CKD-EPI: Chronic Kidney Disease Epidemiology Collaboration
COPD: Chronic obstructive pulmonary disease
CrCl: Creatinine clearance
CVD: Cardiovascular disease
CYC: Cyclophosphamide
eGFR: Estimated glomerular filtration rate
ENT: Ear-nose-throat
FIS: Free-of-infection survival
GC: Glucocorticoids
GPA: Granulomatosis with polyangiitis
HZ: Herpes zoster
MMF: Mycophenolate mofetil
MPA: Microscopic polyangiitis
MPO: Myeloperoxidase
MTX: Methotrexate
PCP: *Pneumocystis jirovecii* pneumonia
PLEX: Plasma exchange
PR3: Proteinase 3
RCT: Randomized controlled trials
RDCI: Rheumatic Disease Comorbidity Index
RTX: Rituximab
SI: Serious infections
VDI: Vasculitis damage index
