# Supplementary material for: Serious infections in ANCA-associated vasculitides in the biologic era: real-life data from a multicenter cohort of 162 patients
Source: Arthritis Res Ther. 2021 Mar 20;23:90. doi: 10.1186/s13075-021-02452-8 (PMC7980356; doi:10.1186/s13075-021-02452-8)
Supplement: Supplementary file 1 — Additional file 1: Suppl. Table 1. Comparison of baseline characteristics according to the prescription of chemoprophylaxis against Pneumocystis jirovecii during the initial induction of the remission (n = 135)*. Suppl. Table 2. Comparison between patients who developed (n = 50) or not (n = 112) serious infections (SI). Suppl. Table 3. Uni- and multi-variate logistic regression analysis of factors associated with serious infections (SI). Suppl. Table 4. Comparison between patients who developed (n = 23) or not (n = 138) serious infections (SI) during the 1st year after diagnosis. Suppl. Table 5. Uni- and multi-variate logistic regression analysis of factors associated with serious infections (SI) during the 1st year after diagnosis. Suppl. Table 6. Comparison of patient characteristics treated with cyclophosphamide (CYC) or rituximab (RTX) based induction regimens. [file 13075_2021_2452_MOESM1_ESM.docx]

**Suppl. Tables**

**Suppl. Table 1.** Comparison of baseline characteristics according to the prescription of chemoprophylaxis against *Pneumocystis jirovecii* during the initial induction of the remission (n=135)*

| **Variable** | **No prophylaxis**  **n=32** | **Prophylaxis**  **n=103** | **p** |
| --- | --- | --- | --- |
| Male, n (%) | 20 (62.5%) | 53 (51.5%) | 0.27 |
| Age, mean (SD) | 63.9 (16.3) | 61.6 (14.2) | 0.43 |
| Age >65 years, n (%) | 20 (62.5%) | 46 (44.7%) | 0.08 |
| MPA diagnosis, n (%) | 16 (50%) | 37 (35.9%) | 0.15 |
| Pulmonary/kidney involvement, n (%) | 19 (59.4%) | 68 (66%) | 0.49 |
| CrCl <30 mL/min, n (%) | 7 (21.9%) | 29 (28.2%) | 0.48 |
| CrCl (mL/min), mean (SD) | 52.7 (29.0) | 53.9 (33.5) | 0.85 |
| BVAS at diagnosis, mean (SD) | 13.5 (5.0) | 13.5 (6.4) | 0.99 |
| CYC+RTX combination therapy, n (%) | 2 (6.3%) | 7 (6.8%) | 0.91 |
| Initial prednisolone dose, mg/day, mean (SD) | 44.6 ± 15.8 | 45.9 ± 14.9 | 0.70 |
| PLEX and/or dialysis, n (%) | 4 (12.5%) | 20 (19.4%) | 0.37 |
| RDCI, median (IQR) | 1 (0-2) | 1 (0-2) | 0.69 |

*n=2 patients were excluded due to missing data

SD: standard deviation, MPA: microscopic polyangiitis, CrCl: creatinine clearance, BVAS: Birmingham Vasculitis Activity Score, CYC: cyclophosphamide, RTX: rituximab, PLEX: plasma exchange, RDCI: rheumatic diseases comorbidity index, IQR, interquartile range

**Suppl. Table 2.** Comparison between patients who developed (n=50) or not (n=112) serious infections (SI)

| **Variable** | **SI (-)**  **n=112** | **SI (+)**  **n=50** | **p** |
| --- | --- | --- | --- |
| Male, n (%) | 55 (49.1%) | 29 (58%) | 0.29 |
| Age, mean (SD) | 59.5 (16.1) | 64.3 (14.6) | 0.07 |
| MPA diagnosis, n (%) | 38 (33.9%) | 22 (44%) | 0.22 |
| **BVAS at diagnosis, mean (SD)** | **11.9 (6.0)** | **14.6 (6.35)** | **0.01** |
| **CrCl (mL/min) at diagnosis, mean (SD)** | **62.9 (34.5)** | **50.9 (36.0)** | **0.048** |
| CrCl <30 mL/min at diagnosis, n (%) | 22 (19.6%) | 16 (32%) | 0.086 |
| Lung involvement, n (%) | 68 (60.7%) | 32 (64%) | 0.69 |
| COPD, n (%) | 8 (7.5%) | 3 (6.4%) | 0.81 |
| CVD, n (%) | 21 (18.8%) | 10 (20%) | 0.85 |
| Diabetes, n (%) | 18 (16.1%) | 13 (26%) | 0.14 |
| **RDCI, median (IQR)** | **1 (0-2)** | **1 (0-3)** | **0.03** |
| **Prednisolone initial dose (mg/d), mean (SD)** | **42.2 ± 13.9** | **48.4** ± **16.9** | **0.01** |
| CYC+RTX* combination at initial induction, n (%) | 4 (3.6%) | 5 (10%) | 0.10 |
| **PLEX and/or dialysis, n (%)** | **10 (8.9%)** | **14 (28%)** | **0.002** |
| Relapse rate, n (%) | 56 (50%) | 29 (58%) | 0.34 |
| Number of relapses, median (IQR) | 0.5 (0-1) | 1 (0-2) | 0.21 |

* Statistically significant differences (p<0.05) between groups are shown in bold

SD: standard deviation, MPA: microscopic polyangiitis, BVAS: Birmingham Vasculitis Activity Score, CrCl: creatinine clearance, COPD: chronic obstructive pulmonary disease, CVD: cardiovascular disease, RDCI: rheumatic diseases comorbidity index, mg: milligrams, CYC: cyclophosphamide, RTX: rituximab, PLEX, plasma exchange, IQR: interquartile range

**Suppl. Table 3.** Uni- and multi-variate logistic regression analysis of factors associated with serious infections (SI)

|  | **Uni-variate analysis** | | **Multi-variate analysis** | |
| --- | --- | --- | --- | --- |
| **Variable** | **ΟR**  **(95% CI)** | **p** | **ΟR**  **(95% CI)** | **p** |
| Male | 1.43  (0.73-2.80) | 0.29 |  |  |
| Age | 1.02  (0.99-1.04) | 0.07 |  |  |
| MPA diagnosis | 1.53  (0.77-3.02) | 0.22 |  |  |
| **PLEX and/or dialysis**  **at diagnosis** | **3.96**  **(1.62-9.72)** | **0.003** | **5.21**  **(1.93-14.07)** | **0.001** |
| CrCl (mL/min) at diagnosis | 0.99  (0.98-0.99) | 0.049 |  |  |
| CrCl <30 mL/min  at diagnosis | 1.92  (0.90-4.09) | 0.09 |  |  |
| Lung involvement | 1.15  (0.58-2.29) | 0.69 |  |  |
| BVAS at diagnosis | 1.07  (1.01-1.13) | 0.014 |  |  |
| CYC+RTX* therapy | 3  (0.77-11.69) | 0.11 |  |  |
| Prednisolone initial dose  (mg/day) | 1.03  (1.002-1.05) | 0.035 |  |  |
| COPD | 0.82  (0.21-3.24) | 0.78 |  |  |
| CVD | 1.14  (0.47-2.76) | 0.76 |  |  |
| Diabetes | 1.65  (0.72-3.75) | 0.23 |  |  |
| RDCI | 1.29  (1.007-1.66) | 0.044 |  |  |
| Relapse | 1.38  (0.70-2.70) | 0.35 |  |  |
| Number of relapses | 1.24  (0.89-1.73) | 0.20 |  |  |

* Statistically significant differences (p<0.05) between groups are shown in bold

SI: serious infections, OR: odds ratio, CI: confidence interval, MPA: microscopic polyangiitis, PLEX: plasma exchange, CrCl: creatinine clearance, BVAS: Birmingham Vasculitis Activity Score, CYC: cyclophosphamide, RTX: rituximab, mg: milligrams, COPD: chronic obstructive pulmonary disease, CVD: cardiovascular disease, RDCI: rheumatic diseases comorbidity index

**Suppl. Table 4.** Comparison between patients who developed (n=23) or not (n=138) serious infections (SI) during the 1^st^ year after diagnosis

| **Variable** | **SI (-)**  **(n=138)** | **SI (+)**  **(n=23)** | **p** |
| --- | --- | --- | --- |
| Male, n (%) | 69 (50%) | 14 (61%) | 0.33 |
| Age, mean ± SD | 59.7 ± 15.8 | 68.2 ± 13.6 | 0.016 |
| MPA diagnosis, n (%) | 48 (34.8%) | 12 (52.2%) | 0.11 |
| **PLEX and/or dialysis, n (%)** | **15 (11%)** | **9 (39%)** | **<0.001** |
| **CrCl at diagnosis, mean** ± **SD** | **62.5** ± **34.4** | **40.6** ± **36.1** | **0.006** |
| CrCl <30 ml/min at diagnosis, n (%) | 29 (21%) | 9 (39%) | 0.058 |
| Lung involvement, n (%) | 82 (59.4%) | 17 (74%) | 0.18 |
| **BVAS at diagnosis, mean** ± **SD** | **12.1** ± **6.2** | **16.7** ± **5.5** | **0.001** |
| **CYC+RTX* combination at initial induction, n (%)** | **4 (3.7%)** | **4 (19%)** | **0.008** |
| **Prednisolone initial dose (mg/day), mean** ± **SD** | **42.5 ± 13.9** | **51.8 ± 18.5** | **0.007** |
| COPD, n (%) | 8 (5.8%) | 3 (13%) | 0.21 |
| CVD, n (%) | 22 (15.9%) | 5 (21.7%) | 0.49 |
| **Diabetes,** n (%) | **20 (14.5%)** | **10 (43.5%)** | **0.001** |
| **RDCI, median (IQR)** | **1 (0-2)** | **2 (1-3)** | **0.01** |

**n=161, one patient excluded.**

*among patients with generalized disease

* Statistically significant differences (p<0.05) between groups are shown in bold

SI: serious infections, SD: standard deviation, MPA: microscopic polyangiitis, PLEX: plasma exchange, CrCl: creatinine clearance, BVAS: Birmingham Vasculitis Activity Score, CYC: cyclophosphamide, RTX: rituximab, mg: milligrams, COPD: chronic obstructive pulmonary disease, CVD: cardiovascular disease, RDCI: rheumatic diseases comorbidity index, IQR: interquartile range

**Suppl. Table 5.** Uni- and multi-variate logistic regression analysis of factors associated with serious infections (SI) during the 1st year after diagnosis

| **Variable** | **Univariate analysis** | | **Multivariate analysis** | |
| --- | --- | --- | --- | --- |
|  | **OR**  **(95% CI)** | **p** | **OR**  **(95% CI)** | **p** |
| Male | 1.55  (0.63-1.83) | 0.34 |  |  |
| Age | 1.04  (1.007-1.08) | 0.019 |  |  |
| MPA diagnosis | 2.04  (0.84-4.98) | 0.115 |  |  |
| **PLEX and/or dialysis** | **5.27**  **(1.95-14.25)** | **0.001** | **3.16**  **(1.001-9.96)** | **0.05** |
| CrCl at diagnosis | 0.98  (0.96-0.99) | 0.008 |  |  |
| CrCl <30 ml/min at diagnosis | 2.42  (0.95-6.14) | 0.064 |  |  |
| Lung involvement | 1.93  (0.72-5.21) | 0.19 |  |  |
| **BVAS at diagnosis** | **1.12**  **(1.04-1.21)** | **0.002** | **1.11**  **(1.01-1.21)** | **0.021** |
| CYC+RTX* combination therapy | 5.6  (1.38-22.7) | 0.016 |  |  |
| Prednisolone initial dose (mg/day) | 1.04  (1.009-1.07) | 0.011 |  |  |
| COPD | 2.42  (0.59-1.89) | 0.22 |  |  |
| CVD | 1.46 (0.49-4.36) | 0.49 |  |  |
| Diabetes | 4.54  (1.75-11.75) | 0.002 |  |  |
| RDCI | 1.66  (1.21-2.29) | 0.02 |  |  |

* Statistically significant differences (p<0.05) between groups are shown in bold

SI: serious infections, OR: odds ratio, CI: confidence interval, MPA: microscopic polyangiitis, PLEX: plasma exchange, CrCl: creatinine clearance, BVAS: Birmingham Vasculitis Activity Score, CYC: cyclophosphamide, RTX: rituximab, mg: milligrams, COPD: chronic obstructive pulmonary disease, CVD: cardiovascular disease, RDCI: rheumatic diseases comorbidity index

**Suppl. Table 6.** Comparison of patient characteristics treated with cyclophosphamide (CYC) or rituximab (RTX) based induction regimens

| **Variable** | **CYC induction**  **(n=99)** | **RTX induction**  **(n=29)** | **p** |
| --- | --- | --- | --- |
| Males, n (%) | 54 (54.5%) | 16 (55.2%) | 0.95 |
| Age, years, mean ± SD | 63.2 ± 14.4 | 60.8 ± 13.0 | 0.42 |
| **MPA diagnosis, n (%)** | **44 (44.4%)** | **7 (24.1%)** | **0.049** |
| BVAS at diagnosis, mean (SD) | 13.0 (6.4) | 13.4 (5.0) | 0.79 |
| Lung involvement, n (%) | 67 (67.7%) | 15 (51.7%) | 0.11 |
| CrCl (mL/min) at diagnosis, mean ± SD | 52.8 ± 30.9 | 60.6 ± 35.4 | 0.26 |
| CrCl <30 mL/min at diagnosis, n (%) | 24 (24.2%) | 7 (24.1%) | 0.99 |
| COPD, n (%) | 6 (6.1%) | 4 (13.8%) | 0.18 |
| CVD, n (%) | 17 (17.2%) | 5 (17.2%) | 0.99 |
| Diabetes, n (%) | 22 (22.2%) | 5 (17.2%) | 0.56 |
| PLEX and/or dialysis, n (%) | 15 (15.2%) | 4 (13.8%) | 0.85 |
| Prednisolone initial dose initial dose (mg/day), mean ± SD | 44.1 ± 14.9 | 48.1 ± 15.4 | 0.24 |

* Statistically significant differences (p<0.05) between groups are shown in bold

CYC: cyclophosphamide, RTX: rituximab, SD: standard deviation, MPA: microscopic polyangiitis, BVAS: Birmingham Vasculitis Activity Score, CrCl: creatinine clearance, COPD: chronic obstructive pulmonary disease, CVD: cardiovascular disease, PLEX: plasma exchange, mg: milligrams
